# How the gut microbiome shapes learning and memory: A comprehensive review

**DOI:** 10.1016/j.ibneur.2025.08.005

**Published:** 2025-08-11

**Authors:** Firoozeh Alavian, Motahareh Safaeian

**Affiliations:** a Department of Biology Education, Farhangian University, PO Box 889-14665, Tehran, Iran; b Bachelor's degree student in Experimental Science Education, Fatemeh Zahra Isfahan Campus, Shahid Rajaee Higher Education Center, Isfahan, Iran

**Keywords:** Microbiome, Gut—brain axis (GBA), Hippocampus, Memory, Learning

## Abstract

Cognitive functions, such as learning and memory processes, are closely related to the gut microbiome. The gut—brain axis (GBA), a complex network of bidirectional communications between the central nervous system and the gastrointestinal tract, plays an important role in regulating these functions. This study aims to investigate the impact of the gut microbiome on learning and memory and to provide new insights into the role of the GBA in these cognitive processes. This narrative review explores various mechanisms through which the gut microbiome affects cognitive functions by reviewing scientific articles related to the gut microbiome, GBA, learning, and memory. The focus is on studies that have investigated the relationship between the gut microbiome, changes in microbial composition, and cognitive functions. The results indicate that the gut microbiome influences brain function and behavior through various mechanisms, such as vagus nerve signaling, effects on the enteric nervous system, the production of neurotransmitters, the regulation of inflammation and the immune system, and the production of metabolites such as short-chain fatty acids (SCFAs). Dysbiosis of the gut microbiota affects hippocampal function, learning, and stress regulation. Additionally, probiotics and prebiotics, along with nutritional status, affect the composition and function of the gut microbiome; therefore, maintaining the balance of the gut microbiome and paying attention to the GBA may lead to improved cognitive functions and the prevention of learning and memory-related disorders. Microbiome-based interventions, such as probiotics and dietary changes, have the potential to increase performance.

## Introduction

**Fig. 1:**
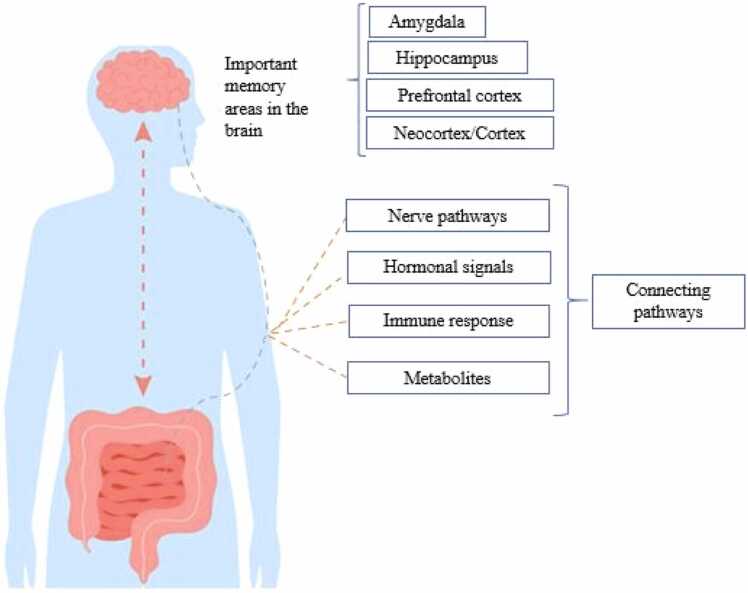
Communication between the brain and gut microbiome.

Learning and memory processes, which are considered to be part of cognitive functions, have traditionally been recognized as unique functions of the central nervous system (CNS). However, existing evidence demonstrates that the gut microbiome (a collection of microorganisms residing in the digestive tract) plays an important role in regulating cognitive functions and learning. This connection occurs via the GBA, which is a complex network of bidirectional communications between the CNS and the digestive system, including neural, hormonal, immune, and metabolic pathways that work in concert to regulate cognitive functions such as memory and learning (Fig. 1) (Bhalla et al., 2024, Qansuwa et al., 2023).

The gut microbiome comprises trillions of microorganisms living in the digestive tract and plays a crucial role in regulating the GBA. In early life, there is a period during which the infant is rapidly growing and developing. Immediately after birth, the microbiome quickly colonizes the infant’s body. During the first few weeks of life, changes in its composition occur, and specific areas of the body are uniquely affected by these microbes. To this end, a study used sequencing methods to analyze stool and saliva samples from 83 children at four time points during the first two years of life, as well as samples from their mothers. The results indicated that the composition of the gut microbiota significantly advances toward a mature microbiome resembling that of adults during the first 2 years of life. The most significant changes in the microbiome composition occurred during the first year of life. The microbiome influences not only physical growth but also the development of the brain and nervous system, such as the hippocampus and amygdala, which are involved in learning and memory. The composition of the microbiota in early life is highly variable and flexible, and as the infant continues to grow, this composition begins to mature. The first major change occurs when the infant stops breastfeeding, leading to alterations in the microbiota population and causing the diversity and number of microbial species to gradually resemble those of adult microbiomes from approximately 2 to 3 years of age in humans. These changes in the microbiota can affect cognitive function and memory throughout life (Wernroth et al., 2022a, Jagodzinski et al., 2019, Moore and Townsend, 2019, Wernroth et al., 2022b).

Microorganisms influence brain function and behavior through various mechanisms, such as vagus nerve signaling, impacts on the enteric nervous system (ENS), the production of neurotransmitters, the regulation of inflammation and the immune system, and the production of metabolites such as SCFAs. These processes directly and indirectly affect cognitive processes, including learning and memory. The normal functioning of the brain is heavily dependent on the microbiome composition. On the other hand, dysbiosis refers to decreased gut biodiversity or an increase in pathogenic bacteria, leading to unhealthy signals being sent to the brain and resulting in inflammation, oxidative stress, energy imbalance, and increased cellular degeneration. These changes affect many aspects of the hippocampus, such as memory and learning, through various pathways, including the vagus nerve, and increase the permeability of the intestinal mucosal barrier and the blood—brain barrier (BBB) (Salami and Soheili, 2022).

Learning and memory are two main processes that play crucial roles in shaping cognition and behavior. These processes depend on hippocampal synaptic plasticity, a part of the brain involved in declarative episodic memory in humans. Episodic memory helps us shape our identity and recall past experiences (Lisman et al., 2017). Disorders in the gut microbiota affect hippocampal function, healthy learning, and stress regulation. Furthermore, prebiotics improve hippocampal-related memory (Papalini et al., 2019), whereas dysbiosis caused by antibiotics reduces hippocampal function (Fröhlich et al., 2016a).

In recent years, extensive research has been conducted on the relationship between the gut microbiome and cognitive functions. A study by Cryan and Dinan (2012) demonstrated that the gut microbiome influences behavior and cognitive functions through the production of neurotransmitters and the regulation of the immune system (Cryan and Dinan, 2012). This study specifically focused on the role of serotonin and GABA in regulating anxiety and depression, both of which are linked to learning and memory. Additionally, Sarkar and colleagues (2016) established that changes in the gut microbiome affect the production of neurotransmitters such as dopamine and serotonin, both of which are involved in learning and memory processes (Sarkar et al., 2016a). This study also revealed that probiotics help improve cognitive function in individuals with mental disorders. In another study, Mayer et al. (2014) reported that the gut microbiome affects brain function through neural and hormonal pathways. This study specifically focused on the role of the vagus nerve in transmitting signals between the gut and the brain. They demonstrated that changes in the composition of the gut microbiome impact stress-related behaviors and memory (Mayer et al., 2014).

In the study by Desbonnet and colleagues (2015), germ-free (GF) mice exhibited deficiencies in memory and learning (Desbonnet et al., 2014). In this study, the gut microbiome was associated with cognitive functions through the production of SCFAs and the regulation of the immune system. The results from other studies indicate that changes in the gut microbiome affect the cognitive functions of individuals with mental disorders such as depression and anxiety. This study specifically focused on the role of systemic inflammation and SCFA production in regulating cognitive functions (Kelly et al., 2016). Foster and McVey Neufeld (2013)) also demonstrated that the gut microbiome can influence cognitive functions through its effects on the immun system and the production of neurotransmitters. This study specifically focused on the role of inflammation and oxidative stress in individuals with cognitive disorders (Foster and McVey Neufeld, 2013).

In another study, Dinan and Cryan (2017) reported that probiotics help improve cognitive functions in healthy individuals and those with mental disorders. This study specifically focused on the role of probiotics in reducing inflammation and improving cognitive functions (Dinan and Cryan, 2017).

This study aimed to investigate the effects of the gut microbiome on cognitive functions, particularly learning and memory, and to provide new insights into the role of GBA in cognitive discussions, especially concerning learning and memory. Given the importance of the gut microbiome in neuroscience and psychiatry research, this article seeks to achieve a better understanding of this complex relationship by examining the various mechanisms through which the gut microbiome affects cognitive functions. Additionally, this article explores the potential role of microbiome-based interventions, such as probiotics and dietary changes, in enhancing cognitive functions.

This article is a narrative review that evaluates the role of the gut microbiome in cognitive functions such as learning and memory. This study provides a comprehensive and analytical perspective on the current state of research in this field by gathering and qualitatively analyzing information from various sources.

## Research method

This article is a narrative review that examines the role of the gut microbiome in cognitive functions such as learning and memory. This study provides a comprehensive and analytical perspective on the current state of research in this field by gathering and qualitatively analyzing information from various sources.

## Results

### The connection between the gut microbiome and brain development

Recent research has shown that there is a close relationship between the gut microbiome and brain development, particularly in areas such as learning, attention, and memory (de Weerth, 2017).

Immediately after birth, microbes rapidly colonize the host’s body. The composition of the microbiome undergoes significant changes in the first few weeks of life and gradually progresses toward a mature microbial community over time. The cessation of breastfeeding and the introduction of solid foods cause a major shift in the microbiome composition. These changes ultimately lead to the formation of a stable microbial community resembling that of adults. Concurrent with the formation of the microbiome, the digestive system is also evolving. The function of the intestinal mucosal barrier, including ion transport and macromolecule permeability, improved with the onset of solid feeding. Additionally, early-life stress, such as when infants are separated from their mothers, delays the development of the intestinal mucosal barrier and increases macromolecule permeability, negatively impacting the development of the digestive system (Moore and Townsend, 2019, Turroni et al., 2020, Fehr et al., 2020, Younge, 2024). The microbiome also plays a crucial role in early human growth, including the maturation of the immune and digestive systems. In the gut microbiome of premature infants, often only a few bacterial species dominate, indicating that host growth processes influence the accumulation of gut microbial communities (Younge, 2024). GBA is highly important for premature infants because of their high sensitivity to dysbiosis. Premature infants are at a high risk of dysbiosis, which is associated with adverse neurological outcomes such as neurodevelopmental disorders, cognitive deficits, and behavioral problems.

The gut microbiome and nervous system evolve simultaneously during early life, making premature infants a unique population, where optimizing gut microbiome colonization has a positive impact on their brain development (Lu and Claud, 2019); the slowest rate of microbiome formation occurs in extremely preterm infants (between 25 and 30 weeks) (Korpela et al., 2018). However, differences in bacterial diversity between preterm infants and full-term infants have not been observed at 4 or 12 months after birth (Claud et al., 2013).

The limbic system, which includes the hippocampus and amygdala, plays a role in regulating emotions and memory storage. The gut microbiome also affects brain development and function through the GBA by influencing the production of neurotransmitters and neurodevelopmental processes (Vaher et al., 2022). The volume of the hippocampus, which plays a crucial role in memory and learning, increases rapidly until the age of two. Neurogenesis (the formation of new neurons) occurs throughout life in specific areas of the brain, including the hippocampus. Reduced neurogenesis early in life may enhance the retention of hippocampus-dependent memories. Because new neurons play a vital role in processing and storing information, when the number of new neurons decreases, the existing neurons in the hippocampus can communicate more effectively with each other, resulting in greater memory retention. In other words, although neurogenesis generally contributes to learning and memory formation, in situations where this process is diminished, existing neurons in the hippocampus may be able to retain information better (Lu et al., 2018, Kim and Shim, 2023, Deng et al., 2010).

Research has shown that disturbances in the microbiota lead to abnormal neurogenesis and affect individuals’ cognitive functions. GF mice show a significant increase in neuron production in the dorsal hippocampal region, which is essential for spatial learning and memory (Mashayekhi and Salehi, 2024). GF mice also exhibit changes in the morphology of the amygdala and hippocampus, indicating the role of the microbiome in the development of brain structures related to memory and learning (Luczynski et al., 2016). Importantly, if mice are recolonized with microbes after infancy, the level of neurogenesis does not return to its natural state. This finding suggests that the regulation of neurogenesis by the microbiota must occur early in life, that is, when the microbiota is being formed and gut physiology is developing; in other words, early life is a critical period in which the microbiota influences brain growth and the production of new neurons (Ogbonnaya et al., 2015). Support for the role of the microbiota in regulating neurogenesis is based on findings that show a decrease in hippocampal neurogenesis due to stress can be prevented by pretreatment with a combination of the probiotics Lactobacillus helveticus and Bifidobacterium longum (Ait-Belgnaoui et al., 2014).

Hippocampal neurogenesis in adults, as shown in mice, is also controlled by signaling from Toll-like receptor (TLR) types, indicating the role of microbes or microbial components in regulating neurogenesis. In the study by Rolls and colleagues (2007), a deficiency in TLR2 reduced neurogenesis and hippocampal volume, whereas TLR4 knockout mice presented increased neurogenesis (Rolls et al., 2007) and decreased learning ability (Okun et al., 2012). Mechanically, the binding of lipopolysaccharide to TLR4 on neural progenitor cells (NPCs) inhibits proliferation and neuronal differentiation through MyD88 signaling, and NF-κB is dependent on PKC α/β (Rolls et al., 2007).

These studies clarify the role of bacteria and bacterial products and their interaction with their respective receptors in the CNS in neurodevelopment and their potential impact on the regulation of cognitive function.

### Relationship between the gut microbiota and brain centers of memory and learning involves interactions among the

The GBA refers to a bidirectional communication network that involves interactions among the central nervous system, the autonomic nervous system, the endocrine system, the immune system, and the gut microbiota. This network allows microbes to interact with the brain; conversely, the brain can also communicate with the gut. Findings show that these microorganisms affect memory, learning, stress, and mood, as well as certain brain disorders (Bhalla et al., 2024, Qansuwa et al., 2023, Gareau et al., 2011). The cognitive function of the hippocampus in rodents and, to some extent, in humans is well established. The hippocampus seems to be particularly exposed to signaling from the microbiota—gut—brain axis related to neuroplasticity, neurogenesis, and neurotransmission (Salami and Soheili, 2022, Moloney et al., 2017). Specifically, the hippocampus plays a role in the formation of short-term and long-term memory, learning, and spatial orientation (Salami and Soheili, 2022).

Since the limbic system and certain other brain centers are involved in both learning and stress regulation and considering the increasing influence of the microbiota on cognitive processes and memory (Salami and Soheili, 2022), the question arises as to how the gut microbiota affects learning and memory mechanisms dependent on relevant brain centers, particularly the hippocampus, and how these effects combine with emotional processing to lead to therapeutic outcomes.

The beneficial impact of the gut microbiota on certain cognitive functions of the brain undoubtedly occurs through effects on the limbic system, particularly the hippocampus. The hippocampus is crucial for regulating complex emotions, learning, and memory storage. This development begins in the early stages of gestation and continues throughout postnatal life (Utsunomiya et al., 1999). The gut microbiota interacts with the hippocampus through several bidirectional pathways. These pathways, which interact and overlap with one another, include the vagus nerve and enteric nervous system (ENS), the metabolism of neurotransmitters or their precursors, such as SCFAs, the hypothalamic—pituitary—adrenal (HPA) axis, and immunity, and the modulation of inflammation.

Multiple studies have shown that the gut microbiota is related to behaviors associated with the limbic system, including learning and memory. Brain imaging in children aged 10–14 years with Crohn’s disease, a type of inflammatory bowel disease (IBD), has shown that these patients experience a reduction in the thickness of the cerebral cortex in specific areas of the brain, including the posterior regions and gyri of the medial frontal lobe. Additionally, the subcortical volume of these patients decreased, and changes in the structure of the limbic pathways (related to emotions and memory) occurred. These changes were also associated with poorer verbal and cognitive memory performance. The authors of this study also identified a connection between inflammation during active disease and cortical thinning, indicating that the greater the degree of inflammation in the body, the greater the likelihood that cortical thinning and decreased cognitive performance are to occur. For example, in children with Crohn’s disease, which is an inflammatory bowel disease, a reduction in the thickness of the cerebral cortex in specific areas of the brain and changes in the structure of the limbic pathways have been observed. These changes are also associated with weaker verbal and cognitive memory performance (Mrakotsky et al., 2016).

The gut—brain axis facilitates communication between the gut and the brain through several mechanisms, including the following:

### Neural pathway

The GBA is a complex communication network that facilitates interactions between the gut microbiota and the CNS. Among the communication pathways of the GBA, neural pathways play an important role in transmitting signals and influencing cognitive functions, learning, and emotional states. This section details the neural pathways, particularly the vagus nerve and the enteric nervous system (ENS), and provides supporting empirical evidence for these interactions.

### Vagus nerve

#### Role of the vagus nerve in GBA communication

The vagus nerve (VN), also known as the tenth cranial nerve (CN X), is one of the most important and functionally significant neural pathways within the GBA and serves as a primary conduit for bidirectional communication between the gastrointestinal tract and the CNS. This nerve is a mixed nerve composed of both sensory (afferent) and motor (efferent) fibers. The vagus nerve originates in the medullary region of the brainstem within the CNS and extends through the thoracic and abdominal cavities, innervating multiple internal organs, including the heart, lungs, and particularly the gastrointestinal tract. Within the gastrointestinal system, vagal sensory fibers form an extensive sensory network that monitors various physiological parameters, such as luminal content, nutrient availability, microbial composition, and immune status. These sensory signals are transmitted to the nucleus tractus solitarius (NTS) in the brainstem, which serves as the first central integration site for peripheral visceral information. These signals relay to higher brain regions critically involved in autonomic regulation, emotional processing, and cognitive function, such as the hypothalamus, amygdala, and prefrontal cortex. In parallel, the efferent branch of the vagus nerve provides parasympathetic motor innervation to the gastrointestinal tract, regulating key physiological functions such as peristalsis, gastric acid secretion, and mucosal immune responses via the cholinergic anti-inflammatory pathway. This bidirectional communication allows the central nervous system to modulate gut function in response to internal and external stimuli, while the gut, particularly through its microbial inhabitants, can influence brain activity, behavior, and cognitive performance. Therefore, the vagus nerve is not merely a passive conduit for signal transmission but rather an active modulator of neurophysiological processes, integrating peripheral signals into central neural circuits that govern learning, memory, and emotional regulation (Srinivasan et al., 2023, Forsythe et al., 2014, Bonaz et al., 2018).

**Fig. 2:**
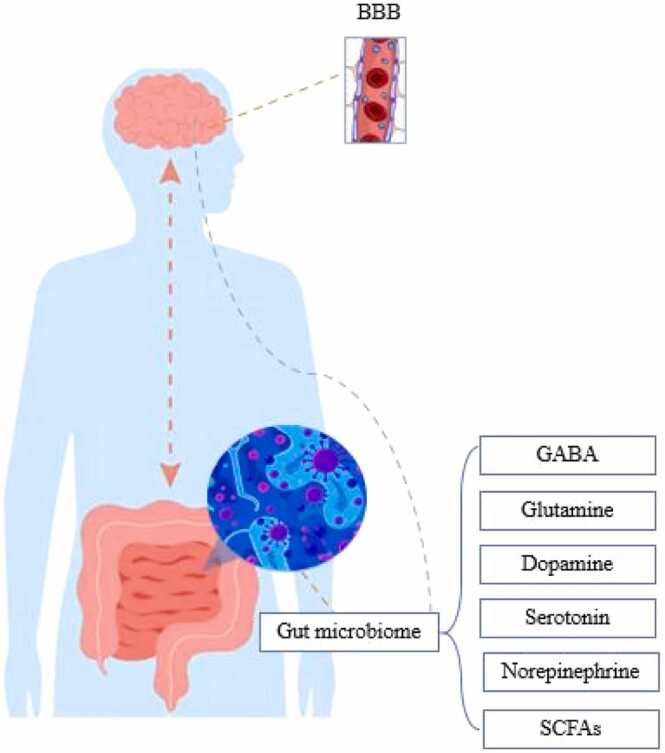
How some substances produced by the gut microbiome connect with the brain. BBB: Blood-Brain Barrier, GABA: Gamma-Aminobutyric Acid, SCFAs: Short-Chain Fatty Acids.

Animal studies have shown that vagus nerve stimulation (VNS) affects various cognitive functions, including learning and attention, alertness, short-term memory, verbal memory recognition, working memory, and decision-making (Olsen et al., 2023); in one study, electrical stimulation of the vagus nerve in mice led to memory consolidation, enhanced learning ability, and retention of new information (Childs et al., 2015). These improvements are associated with changes in the levels of neurotransmitters such as gamma-aminobutyric acid (GABA), glutamate, serotonin, dopamine, and norepinephrine, which help facilitate synaptic plasticity and enhance memory-related processes (Fig. 2) (Olsen et al., 2023).

The gut microbiota potentially may activate vagal afferent nerves (VANs) and influence hippocampal processing. The role of the VN in mediating microbiota’s effects on the hippocampus is underscored by evidence showing that transferring microbiota from feces to GF mice can reverse hippocampal-dependent spatial learning deficits. Moreover, administering the probiotic Lactobacillus rhamnosus (JB-1) to a distinct section of the small intestine enhances VAN activity, a response that requires an intact and functional vagus nerve (Bharwani et al., 2020).

VAN activity is likely altered by the microbiota under normal conditions via enteroendocrine cells. These cells constitute only 1 % of the intestinal epithelium but are among the largest endocrine systems in mammals. Anatomically, these cells are located between the terminals of sensory nerves and the intestinal lumen, where the microbiota resides. They sense the microbiota directly through toll-like receptors (TLRs) and indirectly through metabolites such as SCFAs, nutrients, etc., and transmit this information to VANs (Bonaz et al., 2018, Sternini et al., 2008, Abreu et al., 2005).

#### Enteric nervous system (ENS)

Owing to its extensive network of neurons within the digestive tract, the ENS is referred to as the "second brain" of the body. The neuronal connections of the ENS involve nearly 100 million neurons and are capable of processing information locally within the gut. The gut microbiota also communicates with the CNS through the ENS in the GBA. This bidirectional communication includes neural pathways, endocrine glands, immunity, and hormones that connect the emotional and cognitive centers of the brain with the intestinal functions (Schneider et al., 2019, Sharkey and Mawe, 2023).

Similar to the fate of the vagus nerve, the gut microbiome influences and regulates ENS activity by producing metabolites such as SCFAs, neurotransmitters, and other signaling molecules. These metabolites act as local chemical compounds and play a role in signaling and regulating ENS activity (Balasubramaniam and Srinivasan, 2025). Studies have shown that SCFAs such as acetate, propionate, and butyrate produced from the fermentation of dietary fibers and other carbohydrates by gut bacteria increase neuronal excitability in the ENS. This connection is particularly important due to the GBA, which involves the exchange of signals between the CNS and ENS. Increased neuronal excitability in the ENS also has positive effects on cognitive centers and learning functions (Ray and Mukherjee, 2024, Geng et al., 2022). Studies on GF animals have also shown that bacterial colonization in the gut is essential for the development and maturation of both the ENS and CNS. The lack of microbial colonization is associated with changes in the expression and turnover of neurotransmitters in both nervous systems (Carabotti et al., 2015).

#### Metabolic pathway

The metabolic pathway of the GBA involves the production and release of metabolites and neurotransmitters by the gut microbiota, which influences brain function and behavior. Some of these metabolites include the following:

#### GABA

GABA blocks neural signals from one neuron to another by binding to its receptors. This action reduces the excessive activity of neurons, helps prevent cognitive disorders, and reinforces learning. Increased levels of GABA in the brain also lead to increased synaptic connections between neurons and increased synaptic plasticity, which are essential for learning and memory. Additionally, the GABAergic system influences areas such as the hippocampus and amygdala, which are involved in memory and emotion processing. Vagus nerve stimulation, which involves increasing levels of norepinephrine and GABA in these areas, aids in memory enhancement (Olsen et al., 2023, Chiu et al., 2019). Research has indicated that dietary GABA supplements, when taken with a meal, activate the vagal afferent nerves in mice (Chiu et al., 2019). This finding supports the existence of an alternative pathway through which microbiota-derived GABA affects hippocampal function.

Evidence shows that the VNS facilitates the synaptic localization of GABA A receptors, which is effective in learning and cognitive enhancement (Olsen et al., 2023, Chiu et al., 2019).

#### Glutamine and glutamate

Glutamine is the most common nonessential amino acid in the human body, especially in the gut and muscles. This substance is produced by gut bacteria such as Firmicutes (Ma and Ma, 2019). Additionally, oral glutamine supplementation improves the composition of the microbiota, reduces inflammation, and strengthens the tight junctions of the intestinal epithelium; therefore, a synbiotic supplement (a combination of probiotics and prebiotics) that increases glutamine may be effective in treating dysbiosis and related hippocampal processing disorders (Rao and Samak, 2011).

**Fig. 3:**
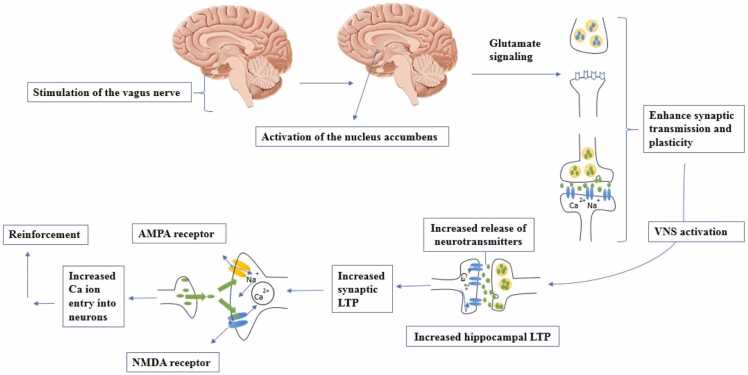
Impact of vagus nerve on increasing ion entry into the neurons and reinforcement of synaptic transmission. VNS: vagus nerve stimulation, LTP: long-term potentiation, NMDA: N-methyl-D-aspartate, AMPA: α-amino-3-hydroxy-5-methyl-4-isoxazolepropionic acid.

Glutamine can be converted to glutamate. The relationship between brain glutamate levels and the VN is crucial for learning processes, cognitive performance enhancement, and memory. Glutamate, the primary excitatory neurotransmitter in the brain and a precursor to GABA, is effective in maintaining synaptic plasticity, which is essential for learning and memory formation. Research has shown that VNS regulates the release of various neurotransmitters, including glutamate. When the vagus nerve is stimulated, specific areas of the brain, such as the NTS, are activated, which in turn affects glutamate signaling in areas related to memory, such as the hippocampus, enhancing the synaptic transmission and plasticity, which are vital for learning. In addition, VNS increases long-term potentiation (LTP) in the hippocampus; LTP strengthens synapses on the basis of patterns of new activity. This strengthening occurs partly owing to increased activity of glutamate receptors, particularly NMDA receptors, which are essential for LTP. The activation of NMDA receptors causes an influx of calcium into neurons, resulting in further strengthening of synapses to facilitate the process of memory consolidation (Olsen et al., 2023, Olsen et al., 2022). The gut microbiome modulates glutamate dynamics by influencing its bioavailability, absorption, and conversion to other metabolites, such as GABA, which affects brain health and neurotransmitter systems. This interaction occurs as part of the GBA and has important implications for neuroinflammation, synaptic plasticity, and cognitive functions such as learning and memory (Fig. 3) (Perna et al., 2019, Gruenbaum et al., 2024).

#### Dopamine

Dopamine is recognized as an important neurotransmitter in the brain's reward system and is closely linked to cognitive functions. This interaction not only affects how learning and decision-making occur but also influences motivation and emotions related to reward seeking. Studies have shown that activation of the VN leads to increased dopamine release in areas such as the ventral tegmental area (VTA) and the substantia nigra, which are essential for neural plasticity related to reward and learning processes, as dopamine is crucial for encoding rewards and guiding behavior (Han et al., 2018, Copas, 2023). Signals from the vagus nerve play a role in shaping how individuals learn from rewards and punishments. Research has demonstrated that VNS affects individuals’ selection accuracy such that people act more slowly when learning from positive experiences, especially when faced with negative feedback. This finding suggests that the vagus nerve may play a role in regulating decision-making processes on the basis of dopamine signals; in other words, the function of the vagus nerve influences how we learn from good and bad experiences (Teckentrup and Kroemer, 2024). Additionally, the vagus nerve transmits internal signals related to metabolic states that affect dopamine levels and, consequently, learning outcomes. For example, in states of hunger or metabolic need, VNS enhances the motivation to learn about food-related rewards. These findings indicate that physiological conditions influence cognitive functions through the regulation of dopamine by the vagus nerve (Olsen et al., 2023, Berthoud and Neuhuber, 2019, Habib, 2024).

Dopamine is connected to the gut microbiome via the GBA, where gut microbes affect dopamine levels through metabolic pathways. Specific bacteria, such as Lactobacillus and Bifidobacterium, modulate dopamine synthesis and metabolism by producing precursors such as tyrosine or influencing neurotransmitter pathways. Furthermore, microbial metabolites and vagus nerve signaling play a role in regulating dopamine activity and affect cognitive functions such as motivation, reward processing, and learning (González-Arancibia et al., 2019, Hamamah et al., 2022, Mhanna et al., 2024).

#### Serotonin

Serotonin is a type of neurotransmitter that plays a role in synaptic plasticity, mood regulation, and cognitive functions. This substance is primarily produced in the gut and activates afferent fibers of the vagus nerve that send signals to the NTS in the brainstem. The NTS integrates serotonergic signals and sends them to higher brain areas that regulate mood and cognitive processes; thus, the vagus nerve acts as a communication pathway between the gut and the brain, facilitating the effects of serotonin produced in the gut on brain functions (Johnson and Wilson, 2018, Bonaz et al., 2019). Research has shown that VNS enhances serotonin signaling in the brain. Additionally, studies have shown that when selective serotonin reuptake inhibitors (SSRIs) are administered, the vagus nerve is activated, which enhances serotonin signaling between the gut and the brain. This interaction indicates that VNS amplifies the effects of serotonin on mood and cognitive function (McVey Neufeld et al., 2019). Furthermore, following vagotomy, the ability of SSRIs to reduce depressive symptoms in animal models decreases, highlighting the importance of vagal pathways in modulating the effects of serotonin (Lee et al., 2025, Siopi et al., 2023). The VN also influences learning outcomes by transmitting signals related to physiological states such as hunger or stress, which affect serotonin levels. Hunger leads to a severe reduction in blood serotonin levels. This reduction in serotonin levels, also known as the happiness hormone, leads to feelings of anxiety, anger, and sadness in individuals, which in turn reduces the level of learning (Breit et al., 2018, Hope, 2023, Armstrong, 2024). Evidence has shown that dysbiosis of serotonin levels in the hippocampus decreases and specifically disrupts hippocampus-dependent spatial memory (Tang et al., 2021). Additionally, Lactobacillus helveticus bacteria in animals under chronic stress restore hippocampal serotonin levels to improve cognitive functions, especially learning and memory, thereby helping to reduce the negative effects of stress on the brain (Yousefzadeh et al., 2020).

#### Norepinephrine

Norepinephrine (NE), a neurotransmitter, plays a significant role in learning and helps improve cognitive performance by increasing alertness and concentration. This chemical is released in response to stress and excitement and aids in regulating arousal and attention in learning and memory processes. The locus coeruleus, an adrenergic center in the brain, plays a crucial role in enhancing memory induced by VNS. The noradrenergic neurons from this area project to the hippocampus, assisting in spatial learning (Olsen et al., 2023, Ross and Van Bockstaele, 2021). These neurons are involved in increasing brain alertness and arousal, thus facilitating learning. The locus coeruleus is primarily recognized as the main source of NE, which is produced from dopamine by the enzyme dopamine beta-hydroxylase in this region. VNS increases NE levels in these areas and enhances synaptic plasticity and LTP, which are associated with learning and memory. Research indicates that VNS enhances memory and learning by increasing alertness and attention mechanisms. This is partly due to the activation of the noradrenergic system, which modulates cortical excitability and memory formation (Olsen et al., 2023). The increase in norepinephrine levels aids in further regulating glutamate signaling pathways in the hippocampus, strengthens synaptic plasticity, and supports learning processes. Notably, the gut microbiota also participates in the production of neuroendocrine molecules such as norepinephrine and adrenaline (Olsen et al., 2022, Dehhaghi et al., 2019).

#### SCFAs

**Fig. 4:**
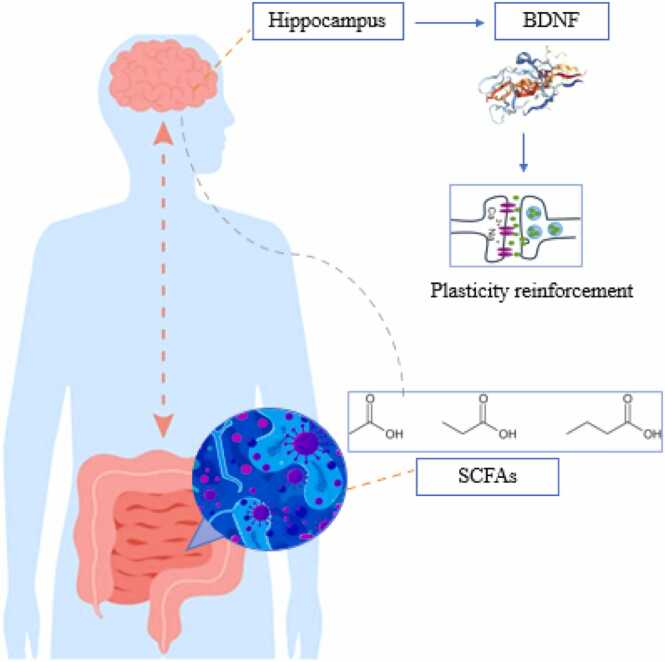
Impact of SCFAs on plasticity reinforcement. SCFAs: Short-chain fatty acids, BDNF: Brain-Derived Neurotrophic Factor.

SCFAs are produced as fermentation products of dietary fibers by gut bacteria such as Firmicutes, Bacteroidetes, Actinobacteria, and Ruminococcaceae (Portincasa et al., 2022). These acids include acetate, butyrate, and propionate, each produced by different species of bacteria. SCFAs influence the CNS by interacting with gut hormones and crossing the BBB. Butyrate specifically enhances BDNF, which is crucial for memory and the growth of neural cells (Vinelli et al., 2022, Silva et al., 2020). Research has shown that decreased BDNF levels are associated with diminished learning and memory since the deletion of BDNF in mice disrupts object recognition and spatial learning (Fig. 4) (Heldt et al., 2007). Butyrate affects histone acetylation and influences the expression of memory-related genes. Additionally, it improves memory performance by enhancing the storage and retrieval of fear memories over a long duration or long-term contextual fear memories through various mechanisms, including effects on the epigenome and the regulation of gene expression. This type of memory is part of the long-term memory that helps us remember fears or defensive reactions to certain environments or conditions (Vinelli et al., 2022, Silva et al., 2020, Peixoto and Abel, 2013, Mohammadi-Farani et al., 2021). Additionally, the anti-inflammatory property of butyrate helps to maintain cognitive function by inhibiting inflammatory factors. Butyrate is particularly important for learning and memory formation because it enhances synaptic plasticity (GdCÁ et al., 2024). Recent studies on GF mice have shown changes in the acetylation of histone lysines. These changes are likely related to mitochondrial dysfunction in the hippocampus, which plays a crucial role in regulating brain function and behavior (Yu et al., 2021). Furthermore, in an interesting study, mice that experienced bilateral carotid artery obstruction presented reduced numbers of beneficial bacteria in the gut and decreased SCFA levels in the brain. This damaged their hippocampal function and gut barrier; however, when beneficial bacteria from the feces of healthy mice were transferred to them, their gut barrier function and SCFA levels in the hippocampus improved, and their cognitive impairments significantly improved (Xiao et al., 2022). Preclinical studies on GF mice also revealed that SCFAs participate in maintaining brain homeostasis and influence learning and cognition. SCFA supplements such as butyrate, acetate, and propionate in drinking water enhance reward-seeking learning behaviors and stress responses in mice (Frost et al., 2014, Zhang et al., 2015).

SCFAs bind to specific receptors in the body, such as GPR41 and GPR43, which play roles in regulating various physiological processes, including metabolism and the immune system. As noted by Liao and colleagues (2022), although these receptors are not found in the brain, they are important for regulating processes related to learning and memory by influencing the nervous system and neuroinflammation. They reported that in mice with sepsis-associated encephalopathy (SAE), the levels of SCFAs such as acetate and propionate were significantly decreased. This reduction was accompanied by an imbalance in the gut microbiome, leading to a decrease in SCFA-producing bacteria. In this study, SCFA injection improved cognitive impairment in these mice and reduced neuroinflammation, but this effect was reversed by a GPR43 antagonist; therefore, GPR43 receptors are crucial for the protective effects of SCFAs against neuroinflammation and the protection of cognitive function in SAE (Liao et al., 2022).

#### Hypothalamic–pituitary–adrenal (HPA) axis

The HPA axis acts as a stress response system that impacts cognitive function and memory. Stress is typically induced by stressors or the absence of immune signaling. The stress response generally involves the lack of inhibition of two main adaptive systems: the fast-acting sympathetic system and the slower-acting HPA, which results in the release of cortisol from the adrenal gland. Cortisol can cross the BBB and exert relatively rapid nongenomic effects as well as slower genomic effects (Joëls and Baram, 2009).

Exposure to the microbiome early in life is essential for the development of the HPA axis. The microbiota influences cognitive performance and memory via stress-related mechanisms. Chronic stress and exposure to cortisol caused by dysbiosis and infection typically disrupts memory and learning (McEwen et al., 2016). The high sensitivity of the hippocampus to changes in the HPA axis is atributted to the high density of glucocorticoid (cortisol) receptors (GRs) in this region. Specifically, hippocampal pyramidal cells, which play a vital role in the consolidation of long-term memories, strongly express GR; however, GR expression is reduced with chronic microbiota depletion (Hoban et al., 2016a). Research has shown that stress decreases microbial diversity and increases the growth of harmful bacteria. When stress disrupts the balance of the gut microbiome, these disturbances can affect signals sent through the vagus nerve and lead to cognitive problems such as reduced learning and memory (McVey Neufeld et al., 2019). Evidence indicates that GF mice exhibit an exaggerated HPA stress response and an altered limbic system, including the prefrontal cortex, hippocampus, and amygdala (Sudo et al., 2004). The Gut microbiota also affects serum glucocorticoid levels. For example, Bifidobacterium supplementation can help reduce chronic stress by lowering blood cortisol levels, thereby improving learning and memory (Tian et al., 2020). Similarly, chronic exposure to glucocorticoids leads to decreased activity in the prefrontal cortex, which is responsible for higher-order thinking and decision-making, whereas activity in the amygdala, which plays a role in emotional responses and survival instincts, increases (Šimić et al., 2021). This change in brain function can disrupt the complex cognitive processes essential for effective learning.

**Fig. 5:**
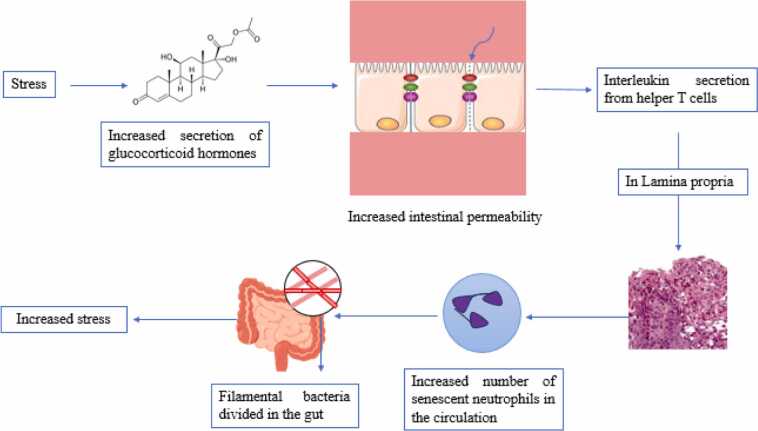
The effect of increased glucocorticoid hormones on increased stress in mice.

In their research, Warren and colleagues (2024) examined the mechanisms linking chronic stress and dysbiosis (imbalance in the gut microbiome) and demonstrated how dysbiosis leads to neurological disorders and impacts mental health. These findings suggest that chronic stress influences the GBA and cognitive functions such as memory and learning through the gut microbiota (Warren et al., 2024). In another study, stress increased the secretion of glucocorticoid hormones, leading to increased intestinal permeability and the release of interleukin-17A (IL-17A) from T helper 17 (Th17) cells in the lamina propria. This process then leads to the expansion of older neutrophils in the bloodstream. Segmented filamentous bacteria in the gut have been identified as essential microbes for the expansion of stress-induced senescent neutrophils, which increase stress in mice (Fig. 5). More importantly, by inhibiting glucocorticoid production, blocking IL-17A, or reducing the gut microbiome that stimulates Th17 cells, stress is alleviated (Xu et al., 2020) and naturally, in these conditions, learning and attention improve.

In the study by Dehhaghi et al. (2019), mice subjected to chronic stress and displaying despair behaviors presented changes in their gut microbiota composition. Notably, the number of beneficial bacteria called Lactobacillus decreased, and the level of a chemical substance called kynurenine increased in the blood. The use of supplements containing Lactobacillus reuteri returned these changes in the microbiota to normal. Interestingly, this treatment not only aided in improving the state of the microbiota but also ameliorated the behavioral abnormalities of the mice. These results indicate that changes in the gut microbiota composition contribute to better metabolic regulation and increased resilience to stress (Dehhaghi et al., 2019); thus, manipulating the microbiota may be an effective way to counteract the negative effects of stress, which is one of the adverse factors in learning.

Recent research has also shown that different types of stress (cognitive stress, stressful life events, and biological stress) have significant effects on the gut microbiome composition. Individuals who have experienced high levels of perceived stress have shown less microbial diversity, which is typically associated with negative health outcomes, including reduced cognitive performance (Delgadillo et al., 2025, Davidson et al., 2018). A comprehensive study conducted on 24,448 older adults indicated that those who experienced high levels of perceived stress were 1.37 times more likely to be at risk for cognitive disorders. Interestingly, these findings remain valid even after accounting for factors such as age and cardiovascular health status. These findings demonstrate that chronic stress not only affects cognitive function and learning but also increases the likelihood of developing more serious diseases, such as Alzheimer’s disease (Kulshreshtha et al., 2023); therefore, stress management and maintaining a healthy gut microbiome contribute to improved cognitive and learning functions.

#### Immune response pathway

The gut microbiome is involved in the development and regulation of the immune system. The relationships among the gut microbiome, immune responses, and learning and memory processes are shaped through complex interactions in the GBA. The gut microbiota and the immune system naturally work together to identify pathogens and trigger an adaptive inflammatory response to restore homeostasis. However, this inflammatory response may extend to the brain, including the hippocampus, leading to cognitive impairments. Brain inflammation is also well recognized in the pathogenesis of cognitive decline (Ray and Mukherjee, 2024, Zheng et al., 2023).

The microbiome interacts with immune cells and impacts both innate and adaptive immunity. Metabolites produced by the microbiome, such as SCFAs, help regulate systemic inflammation and strengthen mucosal immunity. These compounds maintain immune balance by increasing the production of antimicrobial peptides and controlling inflammatory responses; however, during dysbiosis, immune responses become overly activated, leading to chronic inflammation or neuroinflammation and cognitive disorders. Research has shown that changes in the gut microbiota due to the use of antibiotics and infectious agents are associated with alterations in circulating pro-/anti-inflammatory cytokine levels and other neuroactive/immunological substances derived from the gut lumen, which can penetrate the gut mucosa, be transported by the bloodstream, pass through the BBB, and affect the CNS (Petra et al., 2015). This inflammatory cascade negatively impacts learning and memory by disrupting neuronal circuits and accelerating neuronal degeneration.

Studies have indicated that changes in microbiota composition can create immune problems early in life, leading to long-term effects on hippocampus-dependent memory and learning processes. Consequently, mice with immune deficiencies exhibit impaired hippocampus-dependent learning, which can be improved with the use of probiotics early in life (Smith et al., 2014). Interestingly, breast milk is not recognized as a sterile fluid but has a unique microbiome. Breast milk plays a crucial role in inoculating the infant's gut with beneficial bacteria after birth, which is essential for developing a healthy gut microbiome. These beneficial bacteria in breast milk contribute to the infant’s immunity by producing antimicrobial compounds, preventing pathogenic bacteria from attaching to the gut epithelium, and increasing gut mucus production. The origin of these bacteria is still under discussion, but the entero-mammary route (the transfer of microbes from the mother’s gut to the mammary gland) is a possible explanation. The oligosaccharides found in breast milk have a bifidogenic effect, meaning that they promote the growth of Bifidobacterium species, which are considered beneficial for infant immunity. Recent research has focused on isolating potential probiotic strains from breast milk, primarily *Bifidobacteria* and *Lactobacillus*, to improve the immune system, reduce the incidence of infant illnesses, and increase longevity (Lyons et al., 2020).

#### Gut—oligodendrocyte pathway

Oligodendrocytes in the CNS are responsible for producing myelin. The gut microbiota—oligodendrocyte axis is related to the effects of the gut microbiota on the myelination of CNS neurons in the brain. Naturally, changes in myelination in areas involved in memory and learning affect these cognitive functions. The gut–oligodendrocyte microbiota axis influences social behavior, including memory reflections, by regulating myelination in the frontal cortex, which is an important area for predicting multifaceted cognitive behavior and decision-making. Humans experience late myelination of frontal cortex axons in their third decade of life, making them vulnerable to external factors such as microbial metabolites (Ray and Mukherjee, 2024).

Oligodendrocytes have become the focus of research in recent decades because of their vital role in the development, injury, and repair of the nervous system. Recent studies have shown that the gut microbiota during fetal and infant development has a profound impact on central oligodendrogenesis and consequently on subsequent brain injuries. Thus, proper myelination in the CNS depends on the timely availability of the gut microbiota, leading to the differentiation of oligodendrocyte precursor cells (OPCs) into mature and myelinating oligodendrocytes (Mayerl and Heuer, 2023).

#### Experimental evidence linking the gut microbiota to myelination

Luo and colleagues demonstrated that when GF mice were raised with microbiota isolated from rapidly growing neonates, their offspring exhibited increased myelination and less neuroinflammation (Lu et al., 2018). Huban et al. identified myelination genes with upregulated expression in GF mice and demonstrated that hypermyelination is reversed by colonization with normal microbiota after weaning (Hoban et al., 2016b). Interestingly, the use of daily supplements of *Lactobacillus acidophilus* and Bifidobacterium infantis by pregnant mice from day 16 of pregnancy until weaning significantly facilitates OPC growth in their offspring; therefore, maternal probiotic supplementation may provide a safe and effective means of enhancing myelination (Lu et al., 2020).

However, disturbances in the gut microbiome can have the opposite effect. Several preclinical studies have utilized the cuprizone-induced demyelination model to investigate the role of the GBA in myelin pathology. Cuprizone, a copper chelator, selectively induces oligodendrocyte death and demyelination, making it a widely used model for studying multiple sclerosis and other demyelinating disorders (Zirngibl et al., 2022, Vega-Riquer et al., 2019, Steelman et al., 2012). The mechanism of cuprizone-induced demyelination involves two distinct modes: first, ‘intrinsic cell damage’, where mitochondrial dysfunction and impaired myelin protein synthesis directly affect oligodendrocytes; second, ‘extrinsic cellular damage’, in which inflammatory mediators and immune cells such as microglia, astrocytes, and peripheral T cells contribute to oligodendrocyte injury (Zirngibl et al., 2022).

**Fig. 6:**
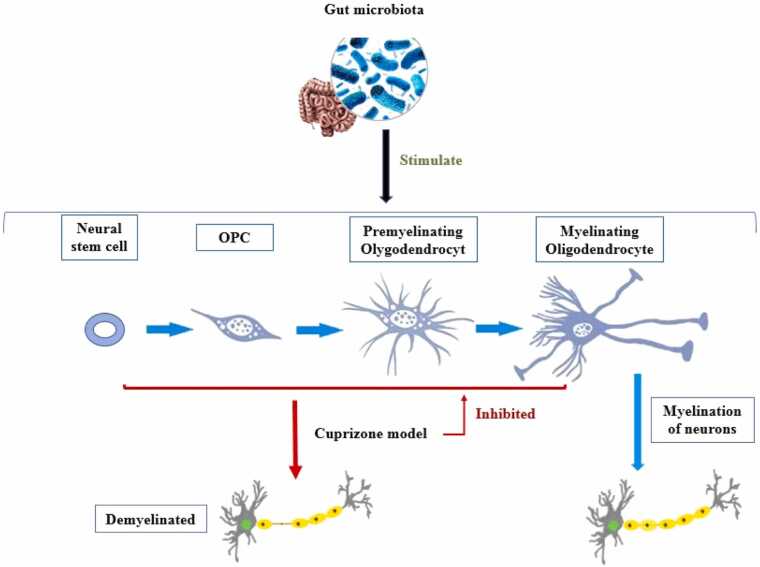
The Role of Gut Microbiota in Oligodendrogenesis, Myelination, and the role of Cuprizone Model. OPC: Olygodendrocyt progenitor cell.

Cuprizone administration in mice results in severe demyelination in specific hippocampal regions, including the stratum lacunosum-moleculare, medial alveus, and CA2/3 areas, which are affected similarly in both young and aged animals (Fig. 6). This demyelination is accompanied by astrocytosis, indicating reactive changes in astrocytes but minimal microglial involvement. Hippocampal damage closely mirrors the demyelination patterns observed in multiple sclerosis (MS) patients, making it a relevant model for studying cognitive deficits in MS. Since the hippocampus plays an important role in learning and memory, this model emphasizes how myelin loss in pivotal pathways can contribute to cognitive dysfunction (Norkute et al., 2009).

In line with these observations, recent evidence suggests that targeting the gut–microbiota–brain axis could be a promising approach to mitigate demyelination and promote remyelination. Wang et al. (2022) demonstrated that the administration of (R)-ketamine in a cuprizone-induced animal model of demyelination reduces demyelination and promotes remyelination processes in the mouse brain. These protective effects are mediated through the activation of the TrkB receptor and a reduction in microglial activity. Most importantly, the results showed that (R)-ketamine was able to shift the composition of the gut microbiota toward a healthier profile and decrease lactic acid levels in cuprizone-treated mice. Furthermore, a significant correlation was observed between the abundance of certain bacterial species, such as *Eisenbergiella massiliensis,* and the extent of demyelination or neuroinflammatory microglial activity in the brain (Wang et al., 2022).

These results clearly show how important maintaining a balanced gut microbiota is for supporting oligodendrocyte development and myelin formation under normal conditions.

#### Dysbiosis of the gut microbiota and impaired myelination

Gut microbiota dysbiosis contributes to impaired oligodendrocyte growth. Alterations in the gut microbiome can modify the microbial metabolome and impact highly permeable bioactive compounds, such as p-cresol, which inhibits oligodendrocyte differentiation and affects the myelination process. The transmission of nerve signals is slower and less efficient, and slower signal transmission impacts the speed and efficiency of cognitive processing, making it more difficult to learn new information or retrieve stored memories (Ray and Mukherjee, 2024, Gacias et al., 2016).

Importantly, the GBA also plays a significant role in pathological demyelination. Bailey et al. (2011) demonstrated that the resident gut microbiota is essential for the induction of in vitro autoimmune encephalomyelitis (EAE), a disease model of multiple sclerosis. GF mice are resistant to EAE unless colonized with polysaccharide-degrading bacteria that enhance inflammatory T-cell responses (Berer et al., 2011). These findings suggest that certain gut microbes can trigger autoimmune responses against myelin. Similarly, antibiotic treatment reduces EAE severity, whereas reintroduction of filamentous bacteria restores pathogenic Th17 cell responses (Ochoa-Repáraz et al., 2009). These results illustrate the impact of gut microbial communities on immune responses that contribute to autoimmune demyelination.

In addition to immune mechanisms, the GBA influences myelin homeostasis via metabolic signaling. Minter et al. (2016) reported that antibiotic-induced disruption of the gut microbiome exacerbated neuroinflammation and altered microglial function, cells essential for maintaining myelin integrity. This study highlighted the systemic effects of microbiota depletion on inflammatory pathways linked to myelin damage, underscoring the importance of a balanced gut microbiome in preserving CNS health (Minter et al., 2016).

Humans experience myelination of the prefrontal cortex (an important brain region for higher cognitive functions) until their third decade of life. This prolonged period of myelinogenesis renders the brain vulnerable to external factors such as microbial metabolites; therefore, dysbiosis during this critical period has long-term effects on cognition (Ray and Mukherjee, 2024, Gacias et al., 2016).

#### Microbial metabolites and myelin regulation

In addition to their direct effects on oligodendrocytes, the gut microbiota influences myelination through their metabolic products. Short-chain fatty acids (SCFAs) such as acetate promote oligodendrocyte differentiation and myelin-related gene expression (Li et al., 2024, Keogh et al., 2021). Conversely, dysbiosis can lead to the accumulation of inhibitory metabolites such as p-cresol, which impair oligodendrocyte function (Gacias et al., 2016). The gut microbiome also regulates microglia, the immune cells of the CNS that support myelin maintenance. GF mice exhibit defective microglial function and abnormal myelin dynamics, which can be restored by microbial recolonization or SCFA administration (Erny et al., 2015).

#### Linking myelin deficits to learning and memory impairment

Myelin integrity is essential not only for rapid neural conduction but also for synchronizing brain networks involved in cognition. The prefrontal cortex and hippocampal connectivity, both heavily dependent on myelinated axons, are crucial for working memory, spatial learning, and emotional memory processing. Disruptions in myelination, whether caused by inflammation, microglial dysfunction, or metabolic insufficiency, have direct consequences on synaptic plasticity and network stability, impairing cognitive performance. Disrupted myelination in cognitive regions such as the prefrontal cortex due to dysbiosis or neuroinflammation leads to slower signal transmission, directly impairing information processing speed and memory function (Ray and Mukherjee, 2024, Gacias et al., 2016). Interventions targeting the gut microbiome, including probiotic supplementation or SCFA administration, may have the potential to improve myelination and cognitive performance, although further research is needed to fully understand the mechanisms involved.

#### The role of probiotics and prebiotics in learning and memory

The beneficial effects of gut microbial symbiosis are facilitated by probiotics (live beneficial bacteria) and prebiotics (indigestible fibers that promote the growth of beneficial bacteria). They help restore microbial balance and reduce inflammation, thus improving cognitive function. Probiotics enhance hippocampus-dependent memory by regulating the HPA axis response to stress (Papalini et al., 2019).

**Fig. 7:**
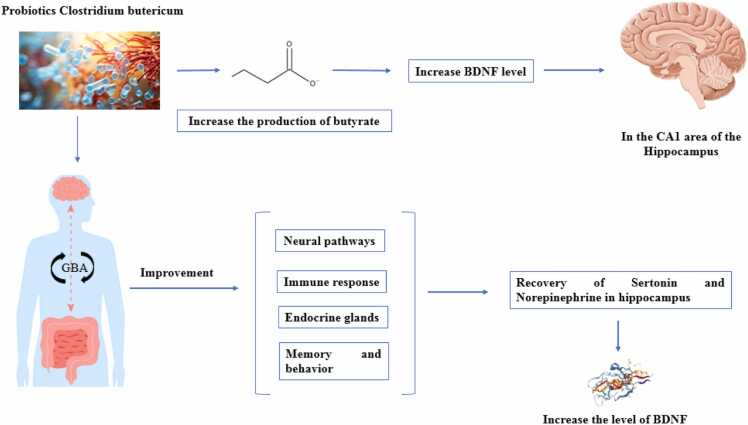
The effect of the probiotic Clostridium butyricum on increasing BDNF. BDFN: Brain-Derived Neurotrophic Factor.

Papalini and colleagues (2019) reported that the consumption of a probiotic supplement for four weeks protects active memory against acute stress disruption and improves hippocampus-dependent learning performance (Papalini et al., 2019). The probiotic Clostridium butyricum aids gastrointestinal health by increasing the production of butyrate, which is an important energy source for gut cells. This probiotic can also increase BDNF levels in the CA1 region of the hippocampus, which is crucial for brain health, memory, and combating cognitive decline (Fig. 7) (Liu et al., 2015). Additionally, the use of a synbiotic that includes a probiotic (*E. faecium*) and a prebiotic (agave inulin) increases butyrate production and improves specific hippocampus-dependent memory and learning performance in mice (Romo-Araiza et al., 2018). In a study by Liang et al. (2015), the consumption of Lactobacillus helveticus NS8 in a chronic stress model improved anxiety- and depression-related behaviors, enhanced cognitive functions, and reduced biochemical disorders. This probiotic showed effects similar to or better than those of the drug citalopram and improved memory and behavior through the GBA by regulating neural, immune, and endocrine pathways. It also restored the levels of serotonin and norepinephrine in the hippocampus and increased BDNF expression (Liang et al., 2015). Furthermore, specific probiotic strains enhance hippocampus-dependent memory by reducing plasma cortisol hormone levels (Bailey et al., 2011). Stress induced by intestinal infection with C. rodentium increases behaviorally induced corticosterone levels. Elevated corticosterone disrupts the expression of c-Fos and BDNF in the CA1 region of the hippocampus, consequently affecting hippocampus-dependent nonspatial memory. Fortunately, probiotics help improve these conditions (Gareau et al., 2011). In the study by Savignac et al. supplementation with Bifidobacterium longum microbiota helped enhance memory consolidation in mice (Carabotti et al., 2015). This microbiota, a type of gram-positive bacteria found in the human gastrointestinal tract, plays a role in the production of lactic acid and acetic acid and helps improve cognitive function while reducing anxiety and depression. Additionally, prebiotics such as inulin and fructooligosaccharides (FOSs) promote the growth of beneficial bacteria such as *Bifidobacteria* and *Lactobacillus*. These bacteria help produce SCFAs, which are effective in improving cognitive performance and protecting against neurodegenerative diseases (Roupar et al., 2022).

Some probiotic strains produce neurotransmitters such as serotonin, dopamine, GABA, and their receptors, which are essential for brain function and learning. Thus, gut dysbiosis early in life leads to reduced expression of the α5 and δ subunits of the GABA-A receptor in the hippocampus and impairment of spatial memory in rodents. Fortunately, these negative effects can be improved by the use of a probiotic combination that includes *Lactobacillus rhamnosus* and *Bifidobacterium longum*(Strandwitz et al., 2019; Liang et al., 2017; Almutairi et al., 2024).

**Fig. 8:**
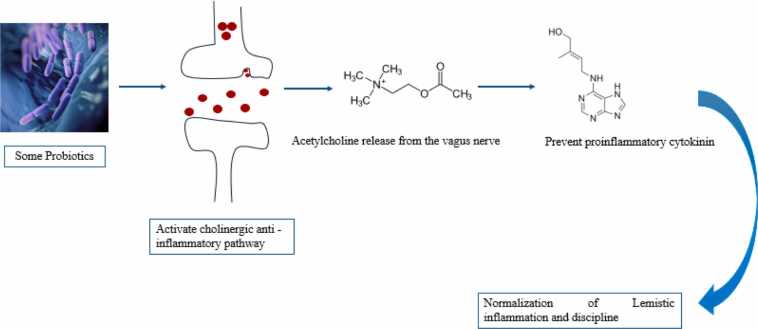
The effect of some probiotics on normalization of lemistic inflammation.

Prebiotics and probiotics reduce systemic inflammation, which is often associated with cognitive decline. Chronic inflammation in the gut results in neuroinflammation, which disrupts synaptic plasticity and cognitive function. Probiotics such as Bifidobacterium have helped reduce inflammatory cytokines, boos the immune system, and improve cognitive outcomes in both animal and human studies (Lyons et al., 2020, Sarkar et al., 2016b). Other studies have shown that some probiotics, particularly Lactobacillus strains, can reduce the levels of corticosterone (CORT) and adrenocorticotropic hormone (ACTH) in the blood. This reduction is linked to improved hippocampal-related spatial memory (Liang et al., 2015). Some probiotics also stimulate the release of acetylcholine in vagal neurons by activating the cholinergic anti-inflammatory pathway. This pathway prevents the release of proinflammatory cytokines and may help normalize systemic inflammation and improve dysbiosis (Fig. 8).

A study on middle-aged mice indicated that the use of synbiotics (a combination of probiotics and prebiotics) improved age-related spatial memory (Han et al., 2022). Memory enhancement was associated with a reduction in proinflammatory cytokines and an increase in BDNF and butyrate levels. Moreover, synbiotics induced changes in the electrophysiological properties of CA1 pyramidal cells in the hippocampus, indicating strengthened synaptic connections, an increase in the N-methyl-D-aspartate (NMDA)/α-amino-3-hydroxy-5-methyl-4-isoxazolepropionic acid (AMPA) receptor ratio, and robust long-term potentiation (LTP) (Romo-Araiza et al., 2018).

Probiotics can also prevent cognitive disturbances caused by a Western diet. For example, the administration of Lactobacillus helveticus to mice fed a high-fat diet improved memory and increased BDNF expression in the hippocampus (Lof et al., 2022), indicating the role of probiotics in enhancing neurogenesis and synaptic plasticity related to learning.

#### The microbiome’s connection to two gastrointestinal diseases related to cognitive function

Many initial studies on the relationship between the microbiome and the brain began by observing cognitive and psychological disorders concomitantly in patients with gastrointestinal diseases. Patients with inflammatory bowel disease (IBD) and irritable bowel syndrome (IBS) often suffer from mood disorders and cognitive deficits in addition to gastrointestinal symptoms. This connection has a significant negative impact on quality of life and complicates treatment strategies (Bernstein, 2016). Next, we explain each of these diseases:

#### Irritable bowel syndrome (IBS)

IBS is a common gastrointestinal disorder characterized by symptoms such as abdominal pain, bloating, and changes in bowel habits (such as diarrhea or constipation). This condition typically presents chronically with periods of flare-ups and remission, and its exact cause is still not fully understood (Camilleri, 2021). While anxiety and depression are the main behaviors observed in these patients, mild changes in cognitive function have also been observed in different subgroups of patients, especially in children and adolescents. Mild verbal memory problems have been reported in patients aged 13–19 with IBD, particularly during the acute phase of the disease (Castaneda et al., 2013). Additionally, in children aged 8–17 years, the administration of the corticosteroid prednisolone, a common treatment for IBD, has led to cognitive deficits compared with patients who are in remission and do not use steroids (Mrakotsky et al., 2013).

Changes in the gut microbiome are proposed as one of the potential causes of IBS. Patients with IBS often suffer from cognitive disorders, including memory issues and information processing problems (Angoorani et al., 2021). Researchers believe that there is a correlation between reduced bacterial diversity in the gut microbiome and the onset of IBS, such that the diversity of gut bacteria in patients with IBS is lower than that in healthy individuals. Furthermore, the abundance levels of certain specific bacterial species differ between IBS patients and healthy individuals (Chung et al., 2016, Pittayanon et al., 2019).

Studies indicate that Fusicatenibacter saccharivorans is associated with changes in the gut microbiota composition in patients with IBS, particularly in individuals who respond positively to probiotic treatments. The abundance of this bacterium significantly increased after treatment, suggesting its potential role in alleviating IBS symptoms by modulating the gut microbiota. Fusicatenibacter saccharivorans likely contributes to gut health by inducing the expression of IL-10, an anti-inflammatory cytokine that suppresses intestinal inflammation (Pang et al., 2021, Tomita et al., 2023). This highlights a mechanism whereby improved gut health can positively affect conditions such as IBS.

**Fig. 9:**
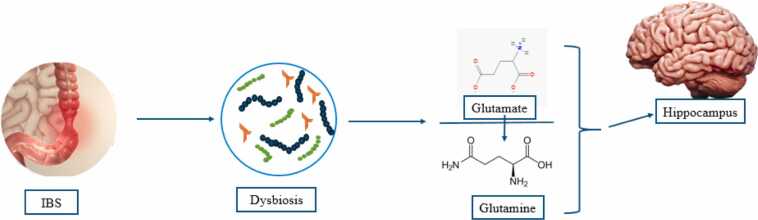
Impact of IBS on Hippocampus. IBS: Irritable Bowel Syndrome.

Research has shown that in individuals with IBS suffering from dysbiosis, the ratio of glutamate to glutamine in the hippocampus decreases. This reduction negatively impacts memory and learning. Conversely, an increase in this ratio is associated with better performance in memory tests (Fig. 9) (Baj et al., 2019).

#### Inflammatory bowel diseases (IBD)

IBD is a chronic inflammatory disease of the gastrointestinal tract that involves a complex interplay of genetic, environmental, and immunological factors. Studies have shown that patients with IBD have reduced diversity in their gut microbiome; a decrease in beneficial bacteria such as *Faecalibacterium prausnitzii* and an increase in pathogenic bacteria such as *Escherichia coli* have been observed (Lopez-Siles et al., 2014).

Dysbiosis and chronic inflammation in IBD negatively correlate with cognitive processes, including learning, through mechanisms involving neuronal inflammation, alterations in neurotransmitter production, and disruption of neurogenesis (Masanetz et al., 2022). Chronic inflammation in IBD leads to the release of inflammatory cytokines such as IL-6 and TNF-α. These cytokines can cross the BBB and contribute to neuronal inflammation (Bonaz and Bernstein, 2013).

If this occurs in learning and memory centers, synaptic flexibility and cognitive function are disrupted. Additionally, changes in the gut microbiota in patients with IBD affect the production of beneficial metabolites such as SCFAs, which are important for maintaining brain health and supporting learning processes; for example, a study by Dong et al. (2019) revealed that, in IBD patients, the biodiversity of the gut microbiota decreases, resulting in reduced production of SCFAs (Dong et al., 2019).

Patients with IBD, particularly children and adolescents, may experience mild cognitive impairments, including verbal memory problems (Castaneda et al., 2013, Masanetz et al., 2022). Specifically, research conducted by Castaneda et al. in 2013 indicated that adolescents with IBD in the acute phase may experience mild verbal memory difficulties. Notably, these individuals made more repeated errors on the California Verbal Learning Test (CVLT) than did their peers with juvenile idiopathic arthritis (JIA). Furthermore, the IBD group exhibited more symptoms of depression than did the JIA group, with approximately one-third of them experiencing at least mild depressive symptoms, particularly during acute illness (Castaneda et al., 2013). These findings suggest that while IBD affects mental health and verbal memory, these effects are generally mild and do not lead to significant cognitive deficits.

**Fig. 10:**
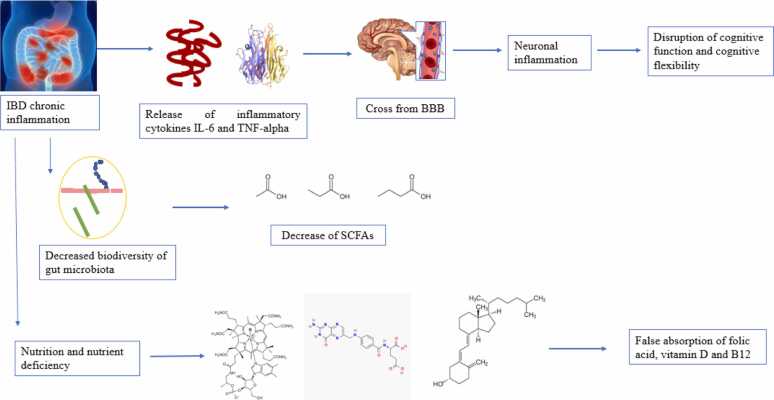
The effect of IBD on Material absorption, memory and learning. SCFAs: Short-chain fatty acids, BBB: Blood-Brain Barrier.

On the other hand, malnutrition and nutrient deficiencies during IBD lead to improper absorption of essential nutrients such as vitamins B12, D, and folic acid (Massironi et al., 2013), which are vital for brain function and cognitive processes (Fig. 10).

#### Relationships among antibiotic use, microbiome, memory, and learning

Research has shown that antibiotics significantly alter the composition of the gut microbiota. For this reason, they are used to create an imbalance in gut microbes. When antibiotics are administered from infancy onward, the number of bacteria and the diversity of the fecal microbiota significantly decrease. These changes have been associated with cognitive problems, as evidenced in tests such as object recognition. Additionally, the levels of certain functional proteins in the hippocampus of adult mice decrease (Fröhlich et al., 2016a).

Whether antibiotics are consumed for a short period or over prolonged and repeated durations, they inflict serious damage to the natural gut microbiome. These alterations disrupt metabolic activities in the gut and can even lead to the emergence of antibiotic-resistant bacteria and pathogenicity, which is particularly concerning in premature infants who are typically treated with antibiotics in the NICU (Lu and Claud, 2019). Owing to their weaker immune system, premature infants are exposed to more stress. This stress renders them more susceptible to infections, necessitating antibiotic treatments. These factors, by exacerbating inflammatory processes in the body and altering the gut microbiome composition, which is essentially the community of bacteria in the gut, affect the neurodevelopment of infants (Maroney, 2003)

Studies have indicated that antibiotic use can have short-term effects on the gut microbiome and cognitive function. Research conducted by Fröhlich and colleagues (2016) revealed that antibiotics cause significant changes in the composition of the gastrointestinal microbiome. These changes are associated with different cognitive behaviors in mice and lead to abnormal levels of body metabolites and alterations in signaling pathways in the brain. Additionally, the amount of antibiotic absorbed in this study was below the detection limit, and the researchers argued that cognitive impairment in antibiotic-treated mice must be due to gut dysbiosis rather than systemic responses from antibiotic administration. In this study, gut dysbiosis was associated with a reduction in metabolites produced by bacteria in the large intestine and changes in the types of fats and molecules produced by microbes in the plasma. Mice treated with antibiotics and subsequently had changes in their gut microbiome did not perform well in memory tests. The antibiotic-treated mice exhibited impaired recognition of novel objects, but spatial memory was not affected. This cognitive deficit is related to specific changes in the expression of signaling molecules associated with cognition, particularly the BDNF factor, the 2B subunit of the N-methyl-D-aspartate (NMDA) receptor, the serotonin transporter, and the neuropeptide Y system, in various brain regions (Fröhlich et al., 2016b).

Understanding the impact of antibiotics on the gut microbiome and cognitive function is crucial for developing strategies to mitigate these effects. Future research should focus on identifying specific bacteria that contribute to cognitive health and finding ways to restore balance to the microbiome after antibiotic use. Additionally, studies on the long-term effects of antibiotic exposure in early life are essential for a complete understanding of its implications for human health. This knowledge helps in creating personalized treatment programs that minimize damage to the microbiome. By addressing these concerns, we can move toward preserving cognitive function and overall health.

## Conclusion

This review underscores the emerging and multifaceted role of the gut microbiome in shaping learning and memory through its interactions with the GBA. We emphasize previously underexplored mechanisms, including microbiota-driven regulation of hippocampal neurogenesis, neurotransmitter systems, and metabolic–inflammatory pathways, that provide a foundation for understanding cognition beyond traditional CNS-centric models.

Our study found that disruptions in the microbial composition during critical developmental stages, especially early in life, can have long-lasting effects on cognitive function. This underscores the importance of safeguarding microbiome integrity from infancy. Additionally, we identified promising therapeutic approaches, including microbiome-targeted interventions such as probiotics and dietary modifications. These strategies demonstrate potential not only in supporting cognitive health but also in mitigating stress-related impairments and neurological complications associated with to dysbiosis.

This work contributes to the growing body of research on the connection between gut health and brain function, highlighting the need for more precise and targeted investigations into how microbial changes can be leveraged to enhance cognitive performance and prevent diseases. In the future, research should focus on examining the long-term effects of microbiome-based treatments, functional differences across various age groups, and the application of advanced multiomics approaches to personalize therapies on the basis of the unique microbial profiles of individuals.

## CRediT authorship contribution statement

**alavian Firoozeh:** Writing – review & editing, Writing – original draft, Supervision, Investigation, Conceptualization. **Motahareh Safaeian:** Writing – review & editing, Writing – original draft, Investigation.

## Ethical approval

N/A

## Informed consent

N/A

## Declaration of Competing Interest

The authors declare no conflict of interest. This article is a review paper and does not involve any human or animal studies requiring ethical approval.

## Data Availability

N/A
